# Farmer and farm worker illnesses and deaths from COVID-19 and impacts on agricultural output

**DOI:** 10.1371/journal.pone.0250621

**Published:** 2021-04-28

**Authors:** Jayson L. Lusk, Ranveer Chandra

**Affiliations:** 1 Department of Agricultural Economics, Purdue University, West Lafayette, Indiana, United States of America; 2 Microsoft Azure Global, Redmond, WA, United States of America; Universitá Cattolica del Sacro Cuore, ITALY

## Abstract

Farmers and farm workers are critical to the secure supply of food, yet this population is potentially at high risk to acquire COVID-19. This study estimates the prevalence of COVID-19 among farmers and farmworkers in the United States by coupling county-level data on the number of farm workers relative to the general population with data on confirmed COVID-19 cases and deaths. In the 13 month period since the start of the pandemic (from March 1, 2020 to March 31, 2021), the estimated cumulative number of COVID-19 cases (deaths) was 329,031 (6,166) among agricultural producers, 170,137 (2,969) among hired agricultural workers, 202,902 (3,812) among unpaid agricultural workers, and 27,223 (459) among migrant agricultural workers. The cases amount to 9.55%, 9.31%, 9.39%, and 9.01% of all U.S. agricultural producers, hired workers, unpaid workers, and migrant workers, respectively. The COVID-19 incidence rate is significantly higher in counties with more agricultural workers; a 1% increase in the number of hired agricultural workers in a county is associated with a 0.04% increase in the number of COVID-19 cases per person and 0.07% increase in deaths per person. Although estimated new cases among farm workers exhibit similar trends to that of the general population, the correlation between the two is sometimes negative, highlighting the need to monitor this particular population that tends to live in more rural areas. Reduction in labor availability from COVID-19 is estimated to reduce U.S. agricultural output by about $309 million.

## Introduction

COVID-19 brought about significant disruptions to the food supply chain [1]. The spike in demand at retail grocery following shutdown orders in March 2020 led to stockouts and empty shelves [2]. The food industry worked to re-allocate output designed and packaged for food service to retail grocery. While some farmers had to plow under crops or dump milk when demand from food service evaporated, others experienced heightened demand, and there was added stress and work at all levels of the food supply chain [3, 4]. While many employees have been able to work remotely during the pandemic, those working in farm, food manufacturing, and grocery were deemed essential [5].

Food and farm workers were declared essential to ensure the public was fed, but this population, which includes many migrant, minority, and low-income individuals, was potentially at high risk for contracting COVID-19. Some of the most notable food system disruptions occurred in the beef and pork packing sectors, which in late April and May 2020, were operating at output levels 40% below the prior year because of plant shutdowns and slowdowns related to the spread of COVID-19 among their workforce [6]. It has been estimated that there were at least 17,000 cases and 91 deaths among U.S. meat and poultry processing workers due to COVID-19 in April and May 2020 [7]. At the farm level, there have been media reports of sizable outbreaks among workers on fruit and vegetable farms [8–10]. These and other events have led some to argue that federal policies have been insufficient to protect worker safety in light of the risks posed by COVID-19 in general [11] and among migrant farm workers in particular [12].

The COVID-19 related risks to the food system have been greatest where labor is most involved. Concerns about food availability and stability are particularly acute in developing countries where labor-intensive traditional value chains exist; however, as the experience with meat packing suggests, there are also agricultural labor concerns in developing countries, particularly for fruits and vegetables that rely on hand planting, weeding, or harvesting [13]. It has been estimated that a mere 40 U.S. counties are responsible for 75% of total fruit and vegetable land farmed [14], indicating the geographic location of COVID-19 cases is particularly important for understanding vulnerability to the food supply. Moreover, given the seasonal nature of agriculture, in which labor is more intensely utilized during planting and harvesting seasons, it is important to understand and characterize the temporal impacts of COVID-19 in those locations where production occurs.

In addition to the direct effect of COVID-19 on agricultural workers, policy and overall economic conditions have affected the supply of migrant agricultural workers. Unemployment rates, which spiked in the wake of COVID-19, affect the demand for migrant labor; the number of H-2A visas issued, which are utilized by migrant agricultural workers, was 4.4% lower April through July 2020 relative to the same time in 2019 [15]. The reductions in migrant labor and other agricultural labor have the potential to adversely affect food supply. Assuming the incidence rate among agricultural workers is the same as that of the general U.S. population, it has been estimated that COVID-19-related reductions in labor through June 30, 2020 resulted in drops in revenue of $16 million, $5 million, and $4 million for lettuce, apples, and grapes, respectively [16].

Despite the high interest in the prevalence of COVID-19 among the agricultural work force, beyond narrow media accounts, there is currently no systematic reporting of such data. The purpose of this paper is to determine the impact of COVID-19 on the farm-level agricultural workforce in the United States. Specific objectives include: 1) estimating the number of COVID-19 agricultural worker cases and deaths over time and across U.S. counties, 2) determining the extent to which locations with larger agricultural workforces are more or less susceptible to COVID-19, and 3) determine the economic impacts of COVID-19-related reductions in agricultural labor on agricultural output.

## Methods

### Estimating COVID-19 cases and deaths among agricultural workers

Data on daily number of confirmed COVID-19 cases and deaths in each U.S. county are obtained from John Hopkins University [17]. Cases and deaths not directly attributable to a county are omitted from this analysis. The exception to this is in Utah, where cases and deaths are reported by region for sparsely populated counties; the Utah cases and deaths not directly attributable to a county were allocated to counties based on each county’s share of the population without direct case counts. Data on population for each U.S. county are obtained from the U.S. Census Bureau [18]. Population estimates for the year 2017 are utilized to match with agricultural labor data.

There are a number of potential data sources on agricultural labor, each with distinct advantages and disadvantages [18]. Given the desire to obtain county-level estimates, and to account for different types of agricultural workers, data from the latest Census of Agriculture in 2017 are utilized [19]. The Census of Agriculture measures four non-mutually exclusive categories of agricultural workers. The first category utilized is that of *agricultural producer*–a category that is often interpreted as a “farmer.” The number of agricultural producers is elicited with the question, “In 2017, how many men and women were involved in decisions for this operation (include family members and hired managers)? Exclude hired workers unless they were a hired manager or family member.” This measure excludes minors but includes men and women who make day-to-day decisions for the operation. In addition, the Census asks about the number of *hired* farm or ranch workers (which could include paid family members and migrant workers who are not paid on contract) in addition to the number of *unpaid* farm or ranch workers (which could include family members). Finally, the Census measures the number of agricultural *migrant* workers (both hired and contract labor), where such worker is defined as one whose employment requires travel that prevents the worker from returning to his/her permanent place of residence the same day. There is likely overlap between the number of producers and the number of unpaid workers: the bivariate, cross-county correlation between these two measures is 0.96. Likewise, there is likely overlap between the number of hired workers and the number of migrant workers; the bivariate, cross-county correlation between these two measures is 0.86. By contrast, the correlation between the number of producers and the number of hired workers is only 0.43. Because of the potential for over-lap between these categories, these measures are not summed in this paper; however, summing the number of producers and hired workers would likely involve minimal double-counting.

Some counties are missing data for particular types of agricultural workers, in which case it is assumed the county has zero of the particular worker type. The Census of Agriculture also reports that some counties have hired, unpaid, or migrant workers, but it does not disclose the exact amount to preserve confidentiality of respondents (confidentiality restrictions do not prevent reporting of number of producers in any county). However, the state-level totals of each labor type are known. Thus, it is possible to determine the number of workers of each type in each state unassigned to a county due to confidentiality. In general, the share of labor unassigned to a county is small. For example, for hired workers, the state with the highest share of workers unassigned to a county was Nebraska, and even in this state, only 1.5% of hired labor was unassigned to a county. The median state had no hired workers unassigned. This is a somewhat bigger issue for migrant workers, where the median state had 9% of total migrant workers unassigned to a county due to confidentiality rules. To address this issue, the state-level labor not directly attributable to a county were allocated to counties with missing labor due to confidentiality based on these counties’ shares of the number of producers among counties with missing data.

There is an important caveat about the labor data. In particular, the labor data consist of counts of the number of people employed on farm operations during the year 2017. As such, there is possibility for double counting if hired or migrant workers work on more than one operation or for more than one employer during the year. Indeed, the Census of Agriculture data indicate 60% of hired labor worked less than 150 days for one operation. However, data from the National Agricultural Workers Survey (NAWS) administered by the U.S. Department of Labor, suggest the potential for double counting is relatively small. Among all hired farm workers, 80% worked for only one employer in the previous 12 months; for migrant workers, 64% worked for only one employer during the year [20]. In 2015/16, the average number of employers a hired farm worker had in the last 12 months was 1.32 [21]. To adjust for the possible double counting, the aggregate estimated number of hired workers and number of migrant workers is reduced by factor of 1.32.

The aforementioned data are merged to estimate the number of COVID-19 cases and deaths among agricultural workers. Let *TCC*_*j*_ represent the total number of confirmed cases of COVID-19 in county *j* and $W_{j}^{k}$ indicate the number of agricultural farm workers of type *k* (*k* = producer, hired workers, unpaid workers, or migrant workers) in county *j*. The expected number of agricultural workers of type *k* confirmed with COVID-19 is $T{CC}_{j}(\frac{W_{j}^{k}}{{POP}_{j}})$, where *POP*_*j*_ is the total population of county *j*. This calculation assumes that agricultural workers in a county contract COVID-19 at a rate equal to that of everyone else in the county, implying the expected number of agricultural workers with COVID-19 is equal to the share of the total population that is an agricultural worker multiplied by the total number of COVID-19 cases in the county.

Total estimated agricultural worker cases in the United States are estimated by summing over all *J* counties in the country: $TCC\_W^{k}=\sum_{j=1}^{J}T{CC}_{j}(\frac{W_{j}^{k}}{{POP}_{j}})$; for hired workers and migrant workers, this calculation is divided by 1.32 to account for the fact that some workers work for multiple employers. Although agricultural worker COVID-19 cases are assumed proportional to the agricultural labor share *within a county*, this relation need not hold at the state or national level. Stated differently, it is not the case that $\sum_{j=1}^{J}T{CC}_{j}(\frac{W_{j}^{k}}{{POP}_{j}})$ is equal to the version of this equation evaluated using national (or state) aggregates: $\left(\sum_{j=1}^{J}T{CC}_{j})(\frac{\sum_{j=1}^{J}W_{j}^{k}}{\sum_{j=1}^{J}{POP}_{j}}\right)$ because of Jensen’s inequality. Agricultural worker deaths are estimated analogously by replacing *TCC*_*j*_ with *D*_*j*_, where *D*_*j*_ are the number of deaths from COVID-19 in county *j*. Total number of deaths for worker type *k*, defined as *D_W*^*k*^, are obtained by summing over all *J* counties.

### Relationship between COVID-19 incidence and agricultural workers

It has been argued that agricultural workers are particularly vulnerable to risks of COVID-19 [12, 22]. However, given the paucity of systematic data on illnesses among agricultural workers, it has been difficult to determine the extent to which COVID-19 incidence has been higher or lower among farm worker populations. To explore this issue, the aforementioned data are utilized to estimate the following relationship:

$$\ln\left(\frac{T{CC}_{j}}{{POP}_{j}}+0.001\right)=\alpha_{0}^{k}+\alpha_{1}^{k}\ln\left(W_{j}^{k}+1\right)+\gamma^{k}S+\varepsilon_{j}^{k},$$ (1)

where the dependent variable is the natural log of the COVID-19 incident rate in county *j* (plus a small number to account for the fact that a few counties have zero cases), ***S*** is a vector of indicator variables for each state, ***γ***^***k***^ is a conformable vector of coefficients, and $\varepsilon_{j}^{k}$ is an error term. The coefficient $\alpha_{1}^{k}$ is relationship between the number of agricultural workers of type *k* and the COVID-19 incidence rate. Given the log-log form of the equation, a 1% increase in the number of workers of type *k* in a county is associated with a $\alpha_{1}^{k}$% change in the incidence of COVID-19 in the county. The same approach is also utilized for death incidence. Similar results are obtained if counties that include no workers or not COVID-19 cases are dropped. Moreover, similar results are obtained if, instead of the log-log specification, a beta regression for proportion data is utilized.

### Economic impacts of reduction in agricultural labor

National accounts on U.S. agricultural productivity from the U.S. Department of Agriculture Economic Research Service [23] are used to estimate the economic impacts of a reduction in agricultural workers. The productivity accounts utilize a growth equation framework relating cost-share weighted changes in quantities of agricultural inputs to the value-share weighted changes in agricultural outputs. In 2017, the last year data were updated, the input cost share represented by hired labor was 0.0779 and the input cost share for self-employed and unpaid family labor was 0.1176. Thus, assuming no changes in productivity during the period in question, each 1% decrease in hired labor and self-employed and unpaid family labor is expected to be associated with a 0.0779% and 0.1176% decrease in total output, respectively.

To utilize the cost-share relationships, the percent reduction in each type of labor from COVID-19 must be estimated. Because the national accounts utilize two types of labor, this analysis uses the percent change in *producers* to reflect changes in self-employed and unpaid labor and the number of *hired* workers to reflect changes in hired labor. Recall *TCC_W*^*k*^ and *D_W*^*k*^ are the total number of estimated COVID-19 cases and deaths associated with worker type *k*. Assuming deaths are also counted as confirmed cases, there are *TCC_W*^*k*^−*D_W*^*k*^ cases which were confirmed but did not result in a death. It is estimated that 40% of cases are asymptomatic [24], leaving (*TCC_W*^*k*^−*D_W*^*k*^)*0.6 symptomatic cases that did not result in death. Let *N*^*k*^ represent the total number workers in the U.S. of type *k*. If each worker works an average of *H*^*k*^ hours per year, then the percent reduction in hours worked from COVID-19 is (2) $\%\Delta H^{k}=[({D\_W}^{k}*S_{D}*H^{k}+({TCC\_W}^{k}-{D\_W}^{k})*0.6**S_{C}*H^{k})/(N^{k}*H^{k})]*100$, where *S*_*D*_ and *S*_*C*_ are the respective shares of average annual hours workers lost from each death and symptomatic case. Note that *H*^*k*^ cancels out of the equation, and this a value need not be assigned. It is assumed that *S*_*D*_ = 1, meaning death results in a complete loss in annual work hours. The symptomatic workers are assumed unable to work some period of time. If workers miss two weeks out of an annual period, their hours worked fall by (2/52) = 3.85%, implying *S*_*C*_ = 0.0385. This is a conservative estimate. If producers do not work full time all year, the denominator would be something less than 52, resulting in a higher share value.

Recall that the cost shares in the national accounts represented by hired labor was 0.0779 and for self-employed and unpaid family labor was 0.1176 [23]. Thus, the estimated loss in agricultural production from COVID is (0.1176*%Δ*H*^*producer*^ + 0.0779*%Δ*H*^*hired workers*^). This value can be multiplied by the value of total agricultural output, which was $451 billion in 2017 [23], to express losses in dollar terms. This calculation does not capture losses that might occur if normal supply chains are adversely affected by worker losses, which might cause delays or bottlenecks that affect food processing and distribution. The calculation also assumes linearity in the relationship between the percent of hours lost and percent change in agricultural output, but if a few illnesses cause entire plants or work crews to be idled, the impacts on output and productivity might be amplified beyond the typical lost marginal productivity of time not worked. These losses also do not include direct costs to workers associated with medical bills or indirect costs associated with “pain and suffering” that might be incurred from COVID-19. Finally, this calculation does not take into consideration the temporal and geographic aspects associated with estimated COVID-19 cases among agricultural workers, but rather relies on aggregate annual national accounting measures. Impacts would be more (less) acute if COVID-19 illnesses and deaths are relatively higher (lower) during planting and harvesting seasons in those locations where large amounts of production occur.

## Results

Table 1 reports the estimated number of COVID-19 cases and deaths among four types of agricultural workers, along with estimated incidence rates for each worker type. As of March 31, 2021, the estimated cumulative number of COVID-19 cases is 329,031 among agricultural producers, 170,137 among hired workers, 202,902 among unpaid workers, and 27,223 among migrant workers. When expressed relative to the total number of each worker type, producers are estimated to have the highest case incidence rate at 9.55% followed by unpaid workers at 9.39%. Table 1 also reports estimated deaths among each type of worker. Death incidence rates vary from 0.179% for producers to 0.152% for migrant workers.

**Table 1:** Estimated number of cumulative cases and deaths from COVID-19 among types of agricultural workers as of March 31, 2021.

Type	Cases	Case Incidence Rate	Deaths	Death Incidence Rate
Producers	329,031	9.55%	6,166	0.179%
Hired Workers	170,137	9.31%	2,969	0.160%
Unpaid Workers	202,902	9.39%	3,812	0.176%
Migrant Workers	27,223	9.01%	459	0.152%

**Fig 1:**
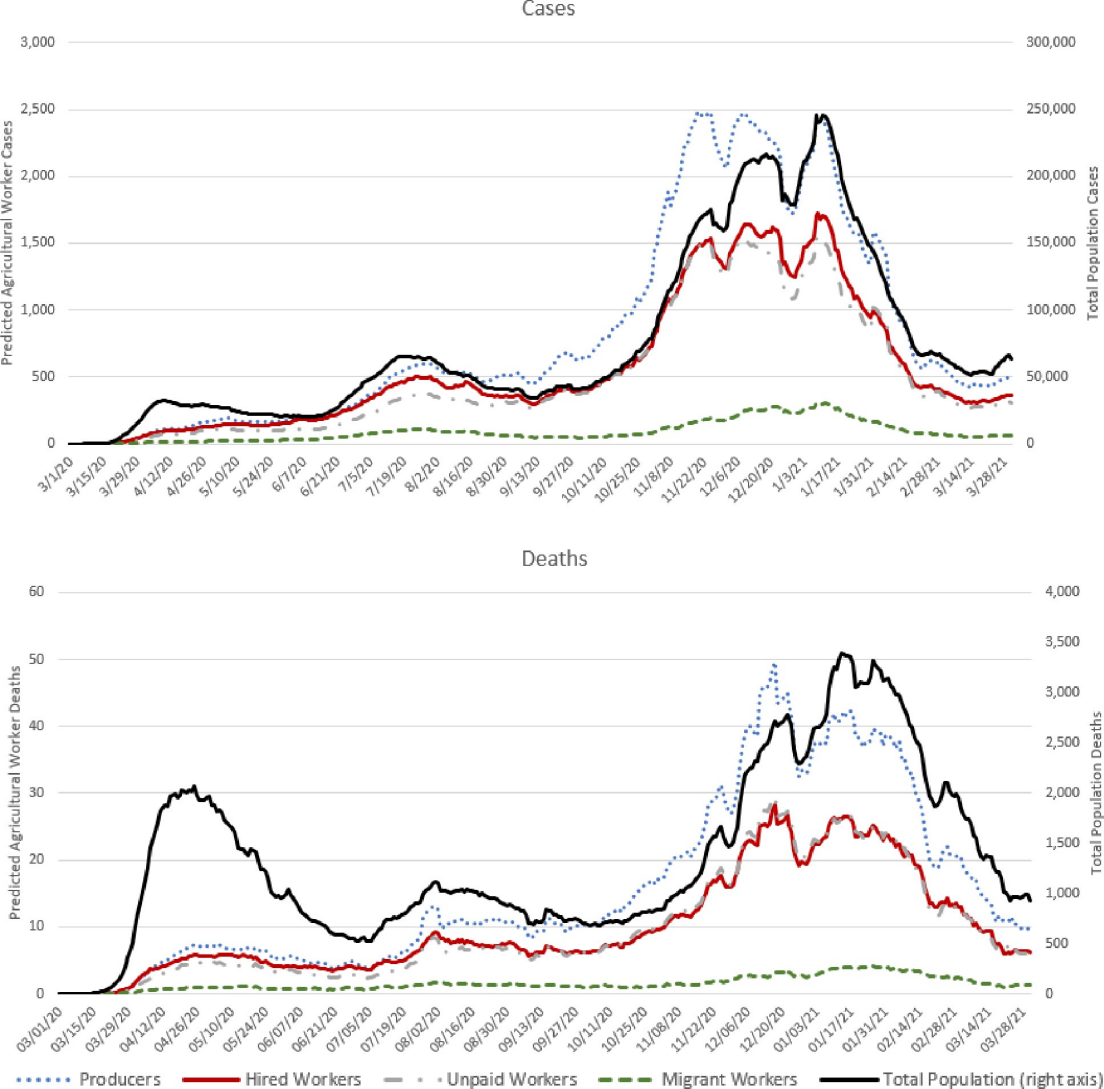
Estimated new daily COVID-19 cases and deaths in United States counties. (the 7-day rolling averages are reported; in the figure reporting total population deaths, outliers on May 18, 2020 (resulting from a few urban New York counties), June 25, 2020 (resulting from a few urban New Jersey counties), and March 12, 2021 were omitted to smooth the trend lines).

**Fig 2:**
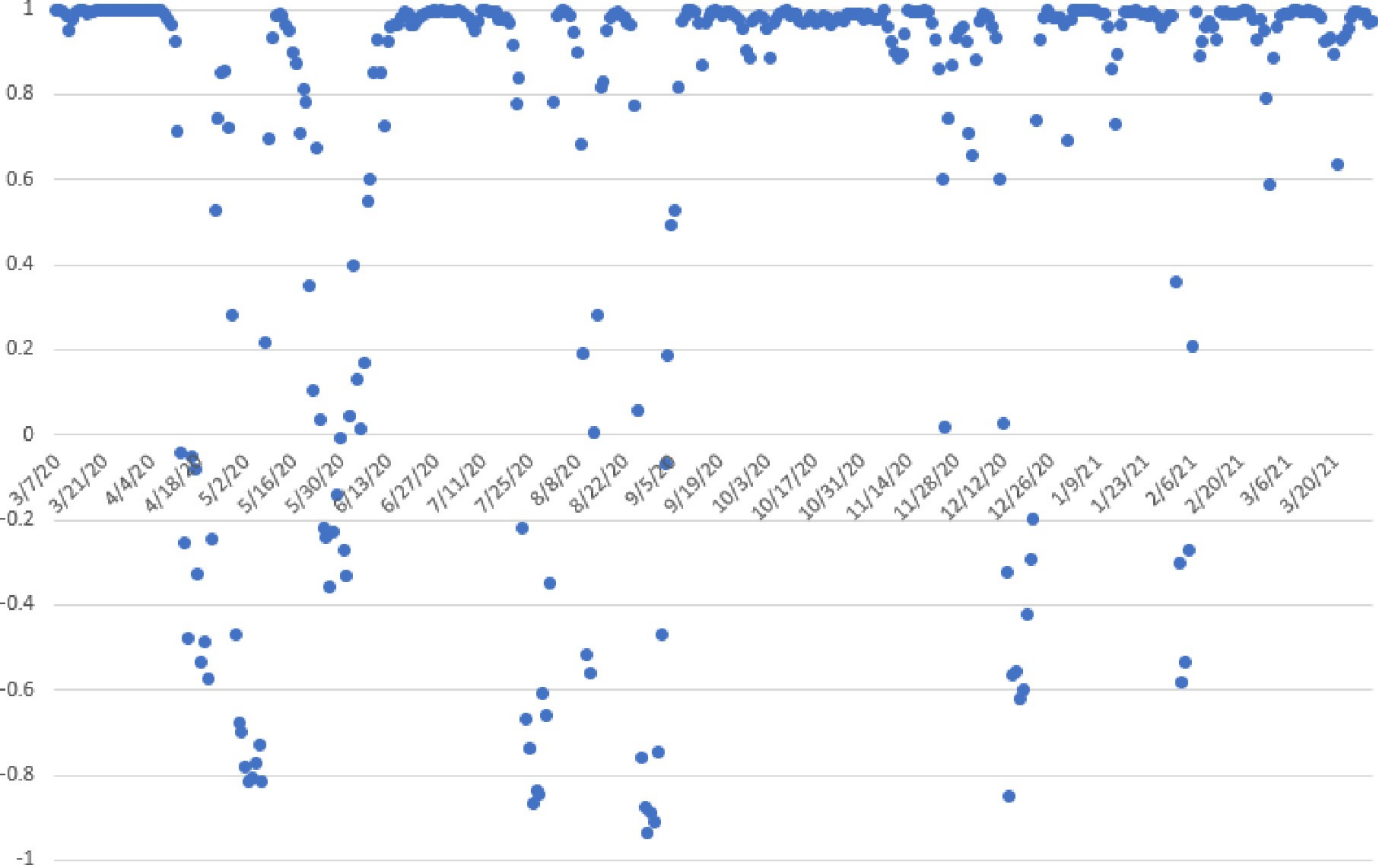
Seven-day rolling correlation between 7-day rolling average of estimated new daily COVID-19 cases among agricultural producers and among the general population.

Fig 1 shows the 7-day rolling average of the daily trends in estimated new COVID-19 cases and deaths for each type of agricultural worker alongside trends in case and deaths in the general population attributable to a U.S. county. Throughout the entire period, trends in agricultural worker cases track closely with those in the general population. However, this is not always the case, as shown in Fig 2. Through April and May, the correlations between new cases in the general population and agricultural workers was negative. While the number of new daily cases among the general population was falling through most of April and May, the number of new cases among agricultural workers was increasing. For example, the correlations during this time period between the total population and producers was -0.36 and the correlation between total population and hired workers was -0.51. The rise in number of farm worker cases during this period coincides, perhaps coincidentally, with the illnesses and shutdowns that occurred in the beef and pork processing sector.

The rate of growth of the number of new daily cases and deaths among agricultural producers throughout October outpaced that of the general population. From the first of July to early November 2020, the ratio of the number of cases among agricultural producers relative to that in the general population steadily increased. On July 1, there were about 0.0076 estimated new agricultural producer cases for every 1 case in the general population; by November 1, there were 0.0155 new agricultural producer cases for every 1 case in the general population, a more than two-fold increase. However, negative correlations between producer and general population cases was observed in December 2020and February 2021, when the number of producer cases began falling more rapidly than in the general population. The trends suggest potentially interesting virus transition dynamics between urban and rural locations, a topic worthy of additional research.

Table 2 reports log-log regressions showing the relationship between the cumulative COVID-19 case and death rates as of March 31, 2021 in each county and the number of agricultural workers in each county. In every case, there is a positive relationship between numbers of agricultural workers and COVID-19 case and death incidence rates indicating counties that tend to have more agricultural workers tend to have higher incidence rates than counties with fewer numbers of agricultural workers. The effects are largest for numbers of hired workers. A 1% increase in the number of hired workers is associated with a 0.04% increase in COVID-19 case incidence and a 0.07% increase in COVID-19 death incidence. Similarly, a 1% increase in the number of migrant workers is associated with a 0.023% increase in COVID-19 case incidence and a 0.041% increase in COVID-19 death incidence. As a case in point, at the end of October 2020, the four counties with the largest number of hired workers at that time (Yakima and Grant, Washington and Fresno, Monterey, and Tulare, California) had an average COVID-19 confirmed case incidence rate of 3.57%. By contrast, among the 77 counties with no hired workers, the COVID-19 confirmed incident rate at the same time was only 2.25%.

**Table 2:** Relationship between cumulative county COVID-19 incidence rate and number of agricultural workers.

	Producers	Hired Workers	Unpaid Workers	Migrant Workers
*Cases*				
Ln(# workers + 1)	0.023*^a^ (0.006)^b^	0.040* (0.005)	0.014* (0.006)	0.023* (0.003)
R^2^	0.43	0.44	0.43	0.44
*Deaths*				
Ln(# workers + 1)	0.054* (0.013)	0.070* (0.012)	0.041* (0.013)	0.041* (0.007)
R^2^	0.34	0.34	0.34	0.34

Note: Each column reports the results of a linear regression in which the dependent variable is either the natural log of the cumulative number of COVID-19 cases or deaths divided by population on March 31, 2021(plus 0.001 for cases or 0.00001 for deaths to include counties that have no COVID-19 cases). Regressions also include state fixed effects.

^a^One asterisk represents statistical significance at the 0.05 level or lower

^b^Numbers in parentheses are standard errors

**Fig 3:**
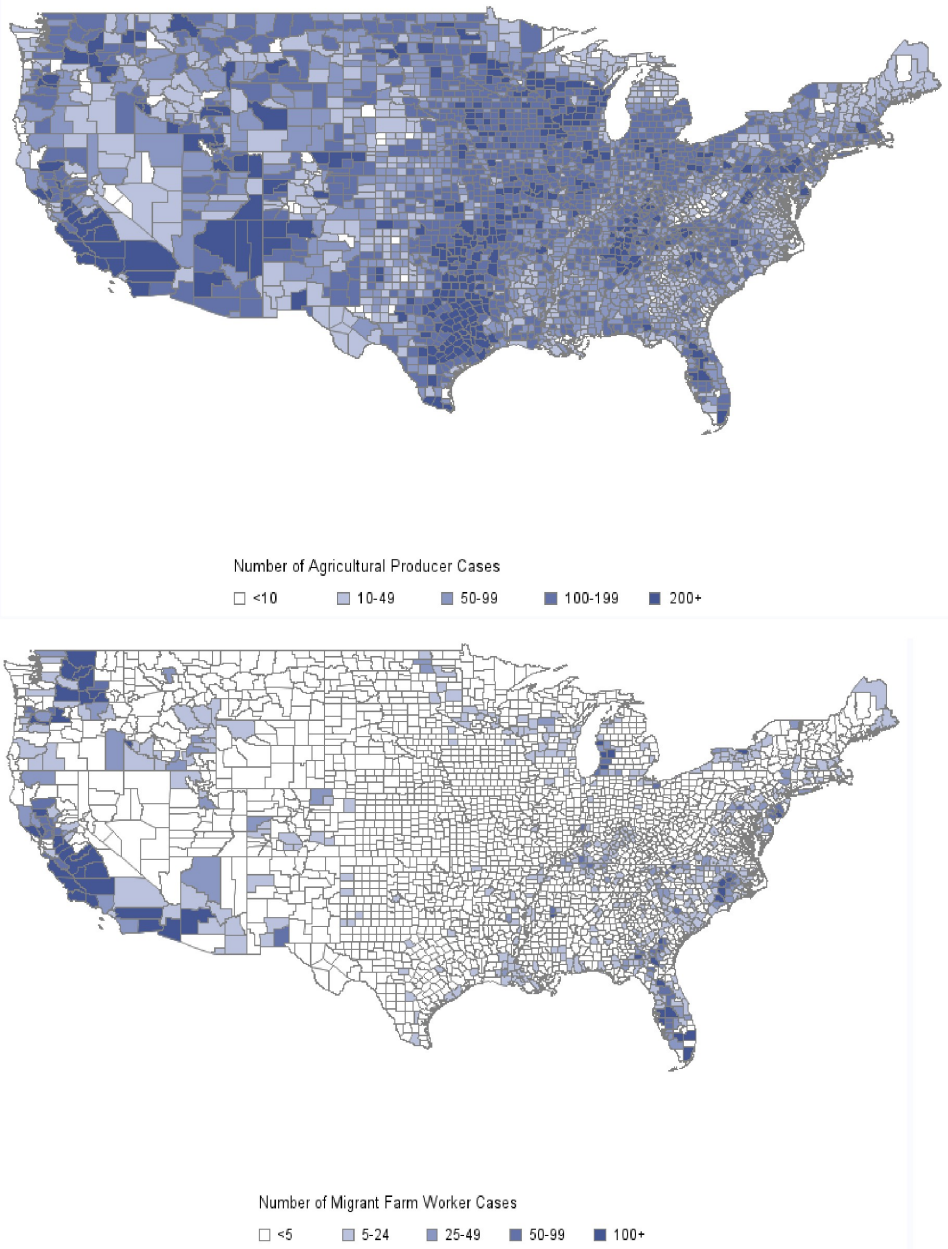
Spatial distribution of cumulative number of estimated cases among agricultural producers and migrant workers by March 31, 2021.

Fig 3 shows the spatial distribution of Cumulative COVID-19 cases among agricultural producers and migrant workers. The highest number of estimated cases among producers is in Arizona (Apache and Navajo counties), Parker TX, California (Fresno, Tulare, and Sand Diego counties), Lancaster, PA, and Miami Dade, FL. The highest number of estimated cases among migrant workers are in California (Fresno, San Joaquin, Tulare, Kern, and Madera counties), Washington (Yakima, Grant, Chelan, and Franklin counties), and Atlantic, NJ.

Estimated COVID-19 cases and deaths can be used to determine losses in labor inputs and agricultural output. Table 1 reports that 329,031 producers are estimated to have had COVID-19 and 6,166 are estimated to have died. Assuming deaths are also counted as confirmed cases, there are 329,031–1,611 = 322,865 cases which were confirmed but did not result in a death. Assuming 40% of cases are asymptomatic (CDC, 2020), there are 322,865*0.6 = 193,719 symptomatic cases. There are a total of 3,447,028 producers in the U.S. Using equation (2), the percent reduction in hours worked among producers COVID-19 is [(6,166+193,719*0.0385)/(3,447,028)]*100 = 0.395%. Similar logic, using the data in Table 1 indicates the percent reduction in hours worked among hired agricultural workers from COVID-19 is 0.283%. Utilizing the respective labor cost shares, the estimated loss in agricultural production from COVID is -(0.1176*0.395 + 0.0779*0.283) = -0.0685%. While this loss is small in percentage terms, it represents a non-trivial reduction in agricultural output. In 2017, total agricultural output was $451 billion (USDA-ERS, 2020b). Reducing this figure by 0.0685% implies a loss of $309 million from COVID-19 through March 31, 2021 resulting from a reduction in agricultural labor inputs.

## Discussion

The emergence of COVID-19 has highlighted the vulnerability of the food supply resulting from losses in farm and agricultural labor. Attempts to ensure a secure supply of food have resulted in policies to declare agricultural workers as essential, which has the potential to put these very workers at heightened risk. Despite these concerns, at present there is scant systematic evidence on the extent to which agricultural workers have been more or less likely to contract COVID-19 than the general population. This research estimates the number of agricultural workers with COVID-19 by relating the number of COVID-19 cases and deaths in each U.S. county with the share of each county’s population comprised of agricultural workers. Results suggest counties that employ more agricultural workers, particularly hired and migrant workers, are at greater risk for COVID-19, findings which suggest these groups are at heightened risk from COVID-19. In addition to the disease risks, hired and migrant agricultural workers represent populations that tend to have toward lower incomes, greater job insecurity, and more perilous immigration and legal status than the general population, which suggest additional relative financial risks resulting from the burden of medical costs or lost time away from work. Beyond the immediate impact on agricultural workers, this research also shows that reductions in agricultural labor have the potential to adversely affect food supply. Over the 13 month period from March 1, 2020 to March 31, 2021, COVID-19 has resulted in an estimated 0.0685% reduction in farm labor input, resulting in an estimated loss of $309 million.
